# Mechanism of Phosphate Desorption from Activated Red Mud Particle Adsorbents

**DOI:** 10.3390/molecules29050974

**Published:** 2024-02-23

**Authors:** Zhiwen Yang, Longjiang Li, Yalan Wang

**Affiliations:** 1 Mining College, Guizhou University, Guiyang 550025, China; yzwyy1011@163.com (Z.Y.); 15809275281@163.com (Y.W.); 2 National & Local Joint Laboratory of Engineering for Effective Utilization of Regional Mineral Resources from Karst Areas, Guiyang 550025, China; 3 Guizhou Key Laboratory of Comprehensive Utilization of Nonmetallic Mineral Resources, Guiyang 550025, China

**Keywords:** red mud, granular adsorbent, desorption mechanism, regeneration

## Abstract

Herein, activated red mud particles are used as adsorbents for phosphorus adsorption. HCl solutions with different concentrations and deionized water are employed for desorption tests, and the desorption mechanism under the following optimal conditions is investigated: HCl concentration = 0.2 mol/L, desorbent dosage = 0.15 L/g, desorption temperature = 35 °C, and desorption time = 12 h. Under these conditions, the phosphate desorption rate and amount reach 99.11% and 11.29 mg/g, respectively. Notably, the Langmuir isothermal and pseudo-second-order kinetic linear models exhibit consistent results: monomolecular-layer surface desorption is dominant, and chemical desorption limits the rate of surface desorption. Thermodynamic analysis indicates that phosphorus desorption by the desorbents is spontaneous and that high temperatures promote such desorption. Moreover, an intraparticle diffusion model demonstrates that the removal of phosphorus in the form of precipitation from the surface of an activated hematite particle adsorbent primarily occurs via a chemical reaction, and surface micromorphological analysis indicates that desorption is primarily accompanied by Ca dissolution, followed by Al and Fe dissolutions. The desorbents react with the active elements in red mud, and the vibrations of the [SiO_4_]^4−^ functional groups of calcium–iron garnet and calcite or aragonite disappear. Further, in Fourier-transform infrared spectra, the intensities of the peaks corresponding to the PO_4_^3−^ group considerably decrease. Thus, desorption primarily involves monomolecular-layer chemical desorption.

## 1. Introduction

Red mud is a porous, alkaline solid material synthesized during alumina production; it exhibits a satisfactory particle size distribution, an average particle size of <0.1 mm, and a specific surface area of ~10–25 m^2^/g [1]. Red mud has satisfactory adsorption characteristics; in particular, it exhibits excellent phosphate adsorption characteristics [2,3]. However, red mud has not been industrially employed for phosphorus adsorption from water, primarily owing to the difficulty involved in phosphorus desorption from red mud particle adsorbents (hereinafter called “red mud adsorbents”), hindering the regeneration and subsequent reuse of the adsorbents. Notably, besides regenerating red mud adsorbents, desorption can also be used for recycling phosphorus resources. Phosphate desorption generally involves using acid, alkali, and salt leaching for phosphate recovery. Simple low-cost salts, such as NaCl and KCl, can be only employed to desorb phosphate from adsorbents exhibiting weak adsorption strengths and nonspecific adsorption [4,5]. High concentrations of such salts generally result in effective desorption [6]; owing to the risk of high salinity, this approach can be problematic if the desorbed phosphate is to be used in crop fertilization and irrigation [5]. Simple salts are ineffective in desorbing phosphate from adsorbents that strongly adsorb phosphate through specific adsorption mechanisms (e.g., ligand exchange and inner-sphere complexation) [7].

As phosphorus adsorption occurs at pH values below 3–4 and above 8–10, acids and bases can be used to specifically and nonspecifically desorb the adsorbed phosphate [8]. At pH values below 3–4, the phosphate adsorption capacity of an adsorbent is low because, at low pH values, phosphate primarily exists as H_3_PO_4_, which is very weakly adsorbed. At pH values above 8–10, adsorbents and the phosphate species in the considered solutions carry highly negative charges (HPO_4_^2−^ and PO_4_^3−^), providing unfavorable adsorption conditions. In addition, the increase in the concentration of OH^−^ with increasing pH increases the competition between phosphate and OH^−^ for adsorption, reducing the phosphate adsorption capacity. If phosphate removal occurs through precipitation at high pH values, as when using Ca and Mg carbonates, then high-pH bases may not desorb phosphate [7,9]. Urano et al. [10] employed activated alumina and Al_2_(SO_4_)_3_ as adsorbents to remove phosphate from water; the adsorbed phosphate was desorbed using NaOH. NaOH treatment resulted in some sulfate desorption and some aluminum dissolution. Therefore, the desorbed adsorbents could not be reused and had to be regenerated by recirculating a solution of Al_2_(SO_4_)_3_ and hydrochloric acid. After alkaline-NaCl treatment, Kuzawa et al. [11,12,13,14] regenerated the adsorbent using 25% (wt%) MgCl_2_ to rebuild the adsorbent structure and restore the adsorption capacity.

In mechanistic studies on phosphorus desorption from red mud adsorbents, acids desorbed phosphorus and concomitantly degraded the adsorbent surface structure, bases reduced the adsorbent adsorption capacity, and salts desorbed phosphate through ion exchange [15,16,17]. Commonly used salts for phosphorus desorption include sodium and potassium chlorides; however, special salts (e.g., NaCl) are sometimes employed to restore the adsorbent structure degraded during desorption. Yaqin et al. [12] studied phosphorus desorption from calcined red mud and reported that the calcination temperature does not substantially affect phosphate desorption. In addition, they reported that the efficiency of phosphorus desorption from the red mud particles was lower in NaOH solutions than in HCl solutions because desorption causes no notable mass loss in the case of alkaline desorbents. However, this mass loss is slightly higher than in the case of deionized water. This is because when using deionized water, compared to physical adsorption, <1% basic desorption occurs and, presumably, because of the involved reverse process of phosphate adsorption [18,19]. For phosphorus adsorption, Mingyang prepared microwave-roasted red mud–fly ash–cement composite granules (85:10:5 mass ratio) using an HCl solution under the following conditions: microwave power = 700 W, roasting duration = 15 min, and roasting temperature = 800 °C. Moreover, they employed a low-concentration NaOH solution for subsequent desorption. Notably, a low-concentration HCI solution is considered a better desorbent, as acids result in the unclogging of adsorbent particles, exposing the effective particle sites for better resorption [20].

Although both acids and alkalis are good desorption agents, the final comprehensive applicability of the employed adsorbent (i.e., after desorption) must be considered to maximize material usage. The results of the present study indicate that, because red mud is alkaline, its desorption using a base increases the risk of introducing high alkalinity in the downstream industrial applications of red mud adsorbents that require a low pH, especially concrete processes. Meanwhile, red mud adsorbents desorbed using acids reduce the adsorbent alkalinity, thus minimally impacting concrete processes. With regard to engineering applications, HCI exhibits good phosphorus desorption from red mud adsorbents; however, the involved desorption mechanism has been little studied. Moreover, if hydrochloric acid is to be used in substantial quantities for such desorption, the involved hydrochloric acid–adsorbent surface interactions and desorption mechanism need to be clarified. Further, the conditions required for optimal desorption (including the hydrochloric acid concentration) must be investigated. The present study is an attempt to address these issues.

Herein, red mud is employed as the primary raw material, sintering-modified charcoal powder is employed as a pore-making agent, and silica sol is employed as a bonding agent. Sintering is used to modify red mud, and static adsorption and desorption tests with HCl solutions of varying concentrations and deionized water are conducted. Finally, surface micromorphological analysis, energy-dispersive X-ray spectroscopy (EDS), mineral composition analysis, Fourier-transform infrared spectroscopy, and fittings of desorption kinetics and thermodynamics are performed to determine the desorption mechanism.

## 2. Results and Discussion

### 2.1. Influence of Different Desorbents on Phosphorus Desorption

**Figure 1:**
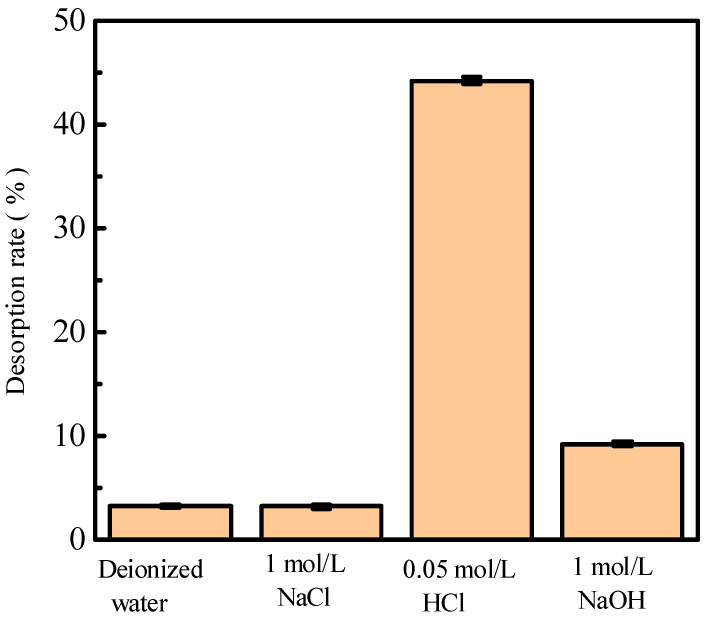
The desorption of phosphorus by alkali (NaOH), acid (HCl), salt (NaCl), and deionized water solutions.

Alkali (NaOH), acid (HCl), salt (NaCl), and deionized water were used to investigate the influence of different adsorbents on phosphorus desorption at a liquid–solid ratio of 0.15 L/g at 35 °C for 18 h. Figure 1 shows phosphorus desorption from activated red mud particles after adsorption. The results indicate that, after 3 h of desorption in deionized water and a NaCl solution, the desorption rate of phosphorus is <10%, which is much lower than those for HCl and NaOH solutions under the same desorption conditions. The phosphorus desorption rate is in the following order: HCl solution > NaOH solution > NaCl solution > deionized water. However, the desorption of the 1.0 mol/L NaOH solution is inconsiderable, which may be due to the insufficient desorption time or desorbent concentration.

### 2.2. Readsorption Performance of Activated Red Mud Adsorbents

**Figure 2:**
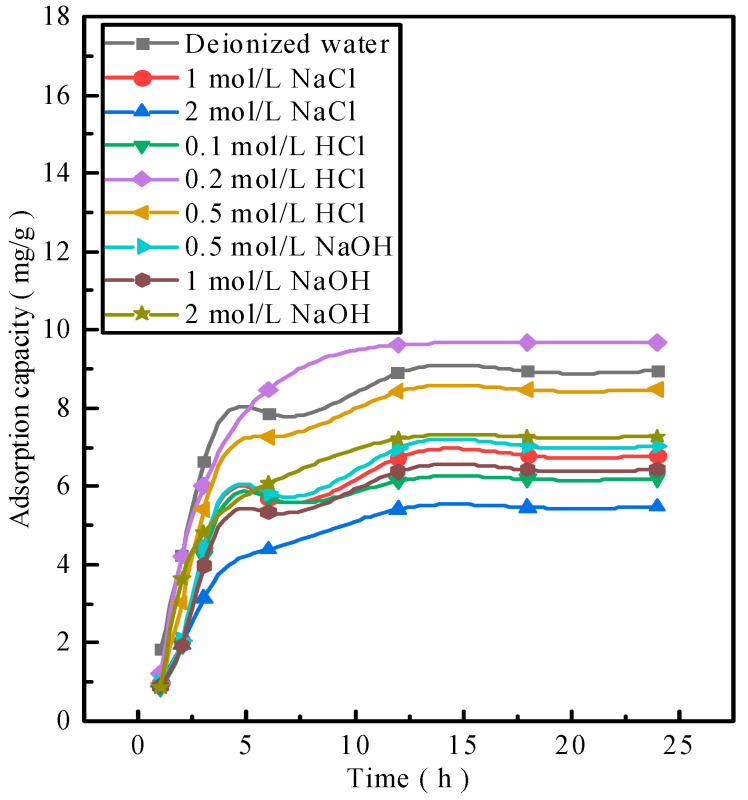
Adsorbent resorption performance.

The activated red mud adsorbents after phosphorus desorption were used to readsorb phosphorus. Notably, 25 g/L of activated red mud adsorbents were injected into a 40 mL conical flask in a phosphorus-containing 300 mg/L solution at a constant temperature of 35 °C to undergo static adsorption for 1, 2, 3, 6, 12, 18, and 24 h. The concentration of phosphorus contained in the solution supernatant was determined through inductively coupled plasma (ICP) emission spectrometry. Figure 2 shows phosphorus resorption by the adsorbent after desorption. The resorption performances for deionized water and 0.2 and 0.5 mol/L HCl solutions are considerably better than those for a low-concentration HCl solution and any concentration of a NaOH solution. Figure 2 shows that 0.2–0.5 mol/L HCl does not destroy the particle structure and even dredges the particle pores, thereby exposing the effective sites inside the particles. This explains why the 0.2–0.5 mol/L HCl solution is better than deionized water. By combining desorption–readsorption and the loss of particles, a 0.2 mol/L HCl solution can serve as a desorbent for desorption and subsequent phosphorus readsorption studies of activated red mud adsorbents.

### 2.3. Effect of Desorbent Dosage on Phosphorus Desorption

**Figure 3:**
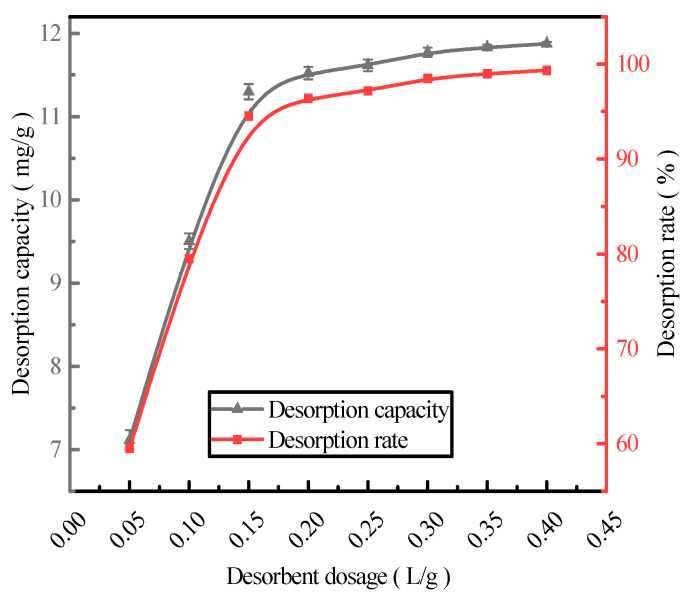
The effect of the desorbent dosage on the phosphorus desorption capacity.

With the HCl desorbent concentration of 0.2 mol/L, phosphorus-containing wastewater at an initial concentration of 300 mg/L was adsorbed by the activated red mud adsorbents, which were then dried at 105 °C for 2 h. The liquid-to-solid ratios of the adsorbents were 0.05, 0.1, 0.15, 0.2, 0.25, 0.3, 0.35, and 0.4 L/g, and static desorption was performed at 35 °C for 12 h. The test was performed at the same time in a conical flask with a liquid-to-solid ratio 1:3 of the adsorbent. The phosphorus concentration in the supernatant of the solution was determined via ICP after the test, and the results were expressed in terms of the amount and rate of phosphorus desorption (Figure 3). The desorption rate and amount of the resorbent leveled off after the liquid–solid ratio exceeded 0.15 L/g. The desorption rate increased to 94.54%, and the desorption amount increased to 11.29 mg/g. The optimal desorbent dosage (0.15 L/g) was employed for phosphorous desorption.

### 2.4. Effect of Initial Adsorption on Phosphorus Desorption

**Figure 4:**
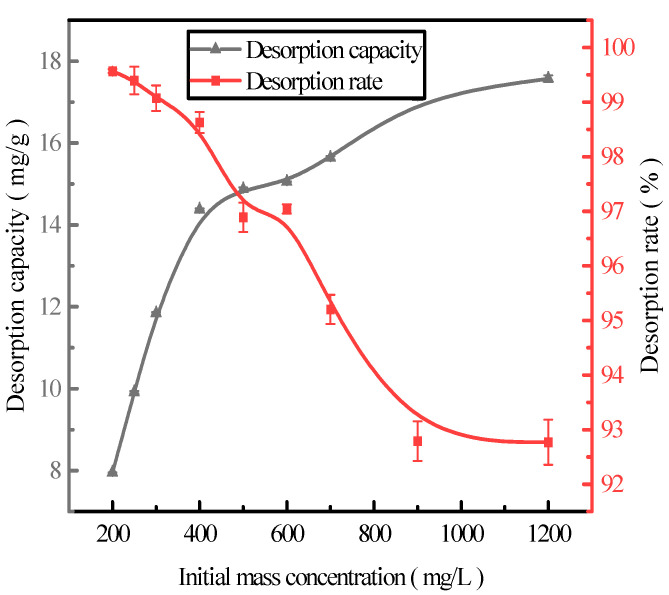
The effect of the initial adsorption on phosphorus desorption.

Here, we discuss the adsorption at the initial concentrations of 200, 250, 300, 400, 500, 600, 700, 900, and 1200 mg/L of activated red mud adsorbents (105 °C for 2 h) and the static desorption of 0.2 mol/L HCl with a desorbent and adsorbent liquid–solid ratio of 0.15 L/g (35 °C in a conical flask for 12 h). Once the phosphorous concentration in the solution supernatant is determined through ICP, the results are based on the amount of desorbed phosphorus. After the test, the phosphorus concentration in the solution supernatant was determined via ICP; the results are expressed in terms of the amount of phosphorus desorbed and the desorption rate in Figure 4. With increasing initial phosphorus concentration, the desorption of the desorbent for the phosphorus adsorbed by the adsorbents increased. The phosphorus desorption was 7.98 mg/g for an initial concentration of 200 mg/L, with a desorption rate of 99.57%, which was primarily attributed to the high adsorption rate. The main reason for this result is that high adsorption rates drove the desorption of the desorbent to the adsorbent, increasing the number of adsorption sites used on the particle surfaces and leading to better desorption. The desorption rate decreased with increasing initial phosphorus concentration, which was consistent with adsorption. The particles presented in Figure 4 show that the desorption rate of phosphorus adsorbed by the adsorbents exceeded 99% when the initial concentration was <300 mg/L. Thus, high-efficiency phosphorus removal was realized.

### 2.5. Effect of the Reaction Time on Phosphorous Desorption

**Figure 5:**
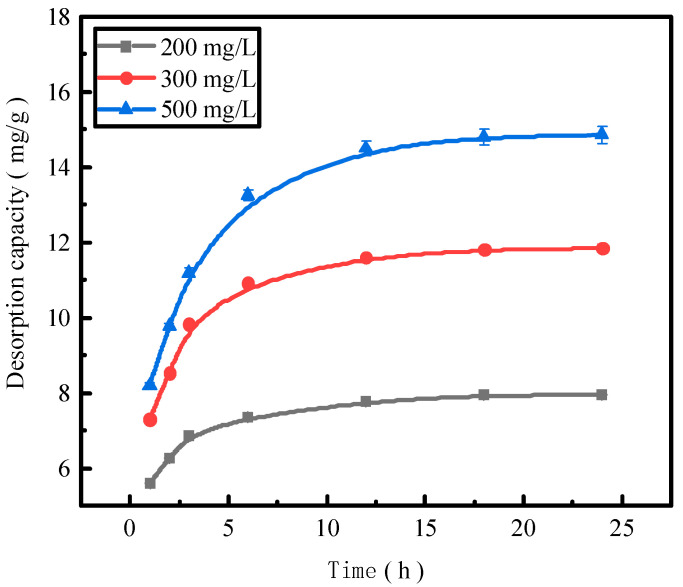
The phosphorus desorption capacity as a function of time.

The reaction time is also an important factor affecting the desorption effect of the desorbent on pollutants. We use activated red mud adsorbent for adsorption at 105 °C (drying for 2 h), a desorbent and adsorbent liquid–solid ratio of 0.15 L/g, and the initial concentrations of 200, 300, and 500 mg/L of the desorbent solution at 35 °C in a conical flask to conduct static desorption for 1, 2, 3, 6, 12, 18, and 24 h. The tests were performed via ICP to determine the effect of phosphorus desorption. After each test, the phosphorus concentration in the supernatant of the solution was determined via ICP, with the results expressed in terms of the amount of phosphorus desorbed (Figure 5).

The trend of the desorption amount of phosphorus adsorbed by the desorbent for the activated red mud adsorbent remained consistent for different initial phosphorus concentrations, and the increase in the desorption amount of the desorbent for an initial solution of 200 mg/L was considerably less than that for the initial concentrations of 300 and 500 mg/L for the first 12 h of the reaction. This result is attributable to the low concentration of phosphorus in the initial phosphorus solution of 200 mg/L, which resulted in less phosphorus adsorbed by the activated red mud adsorbent, in turn increasing the desorption rate. The desorption was lower when phosphorus was adsorbed by the adsorbent and desorbed after 12 h of the reaction under the condition of a low concentration.

### 2.6. Effect of the Reaction Temperature on Phosphorous Desorption

**Figure 6:**
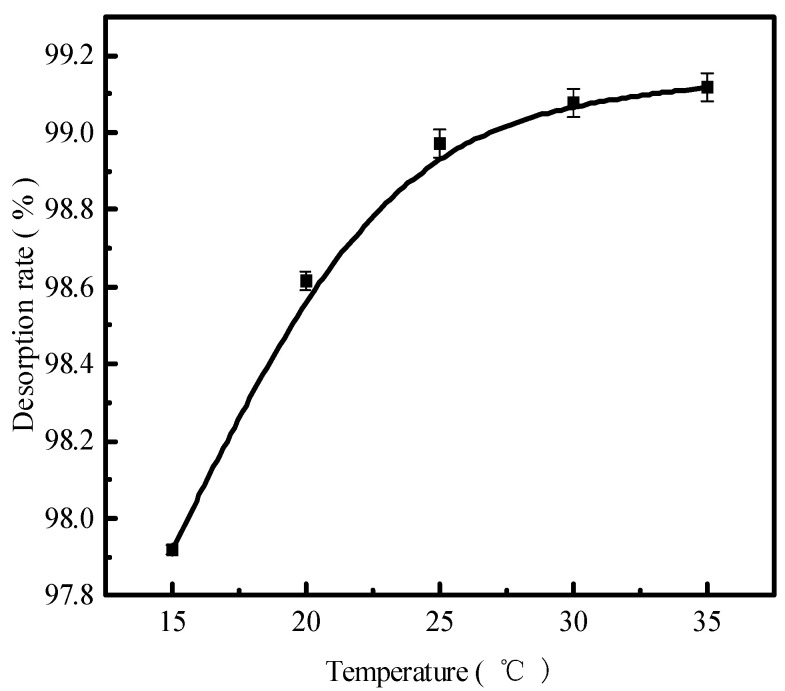
The phosphorous desorption rate as a function of time.

The reaction temperature is also an important factor affecting the desorption effect of the desorbent on pollutants. Figure 6 presents the results in terms of the phosphorus desorption rate. The desorption rate and the amount of desorbent desorbed for a liquid–solid ratio of 0.15 L/g, the initial concentration of 300 mg/L of a 150 mL desorbent solution at 15 °C, 20 °C, 25 °C, 30 °C, and 35 °C for static desorption, and the static desorption in a conical flask for 12 h were determined by employing ICP to obtain the concentration of phosphorus in the solution supernatant. The phosphorus desorption rate increased with increasing reaction temperatures. At 35 °C, the phosphorus desorption rate was 99.11%, and the desorption rate stabilized above 25 °C, indicating that desorption was heat absorbing. The increase in temperature accelerated the intermolecular thermal motion and the diffusion of phosphorus because adsorption and desorption are constantly converted for the reaction to occur. Moreover, desorption is a chemical reaction process in which HCl and the adsorbent surface of phosphate dissolve the other ions while sparing the HCl action channel. This increases the contact area of the reaction and temperature, elevating the chemical reaction rate and, thus, the desorption rate. In addition, according to the previous adsorption behavior study, phosphorus adsorbed on the adsorbent of red mud particles is an attached state, with only hydrogen bonding forces and van der Waals forces acting on it. Consequently, the rate at which phosphate escapes the system will increase as a result of the temperature increase intensifying the molecular movement.

### 2.7. Desorption Isotherm

**Figure 7:**
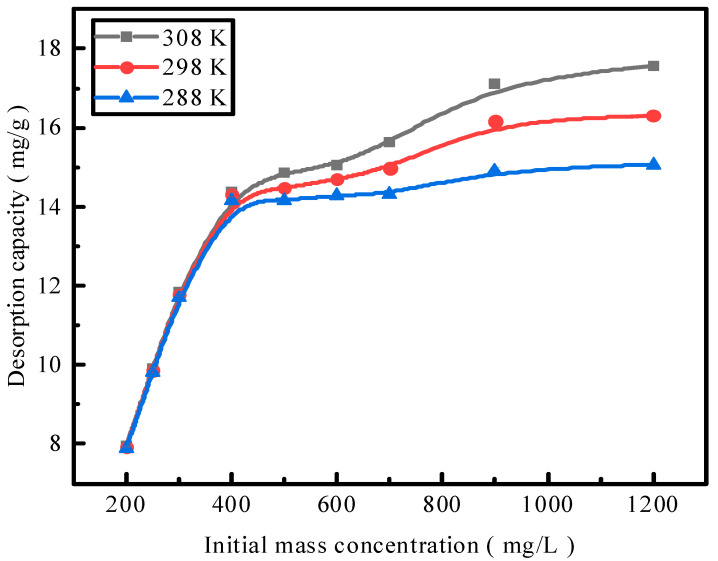
The effect of the reaction temperature on phosphate desorption.

**Figure 8:**
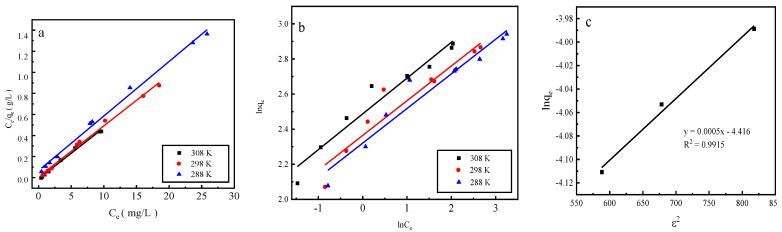
The fits of three desorption isotherm models: (**a**) the Langmuir model, (**b**) the Freundlich model, and (**c**) the D–R model.

Adsorption by activated red mud adsorbent was conducted for the initial concentrations of 200, 250, 300, 400, 500, 600, 700, 900, and 1200 mg/L, and adsorption after drying at 105 °C for 2 h and the desorption of a 0.2 mol/L HCl desorbent were performed at a desorbent and adsorbent solid–liquid ratio of 0.15 L/g and constant temperatures of 15 °C, 25 °C, and 35 °C. Static desorption was performed in a conical flask for 18 h. The desorption effect of the desorbent on phosphorus was investigated at different ambient temperatures, with the results expressed as the desorption rate of phosphorus (Figure 7). The experimental results fit with Langmuir and Freundlich isotherms and a D–R model, and the results of the fits are presented in Figure 8 and Table 1. The Freundlich model explains multimolecular-layer surface desorption, and the Langmuir model describes homogeneous desorption, wherein pollutants are desorbed as a monomolecular layer with homogeneous active sites on the desorbent surface [21].

Desorption is primarily affected by the Langmuir adsorption isotherm model, indicating that monomolecular-layer phosphorus desorption occurs on the surfaces of the red mud particles activated by HCl. The average adsorption free energy |E| obtained using the D–R model is 31.62 kJ/mol, which exceeds 16 kJ/mol, indicating that desorption is chemical.

### 2.8. Desorption Thermodynamic Analysis

**Figure 9:**
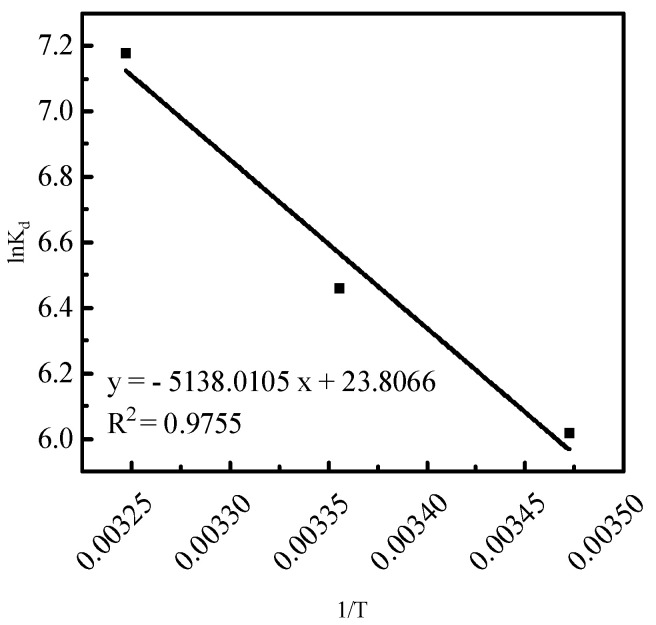
The thermodynamic curves of phosphorus desorption from activated red mud particle sorbents.

To further investigate the effect of temperature on phosphorus desorption, thermodynamic parameters were utilized to reflect the desorption characteristics. The desorption thermodynamic curves of $lnK_{d}$ versus $\frac{1}{T}$ were numerically fitted using Equations (9)–(12) (Figure 9 and Table 2).

In Table 2, $\Delta G^{\theta }$ is <0, indicating that hydrochloric acid is favorable for phosphorus desorption and desorption is spontaneous. Moreover, $\Delta H^{\theta }$ is >0, indicating that phosphorus desorption using hydrochloric acid is a heat-absorbing reaction, suggesting that high temperatures are favorable for desorption. Further, $\Delta S^{\theta }$ is >0, suggesting that the solid–liquid interface degree of freedom of desorption increases.

Notably, $\Delta G^{\theta }$ is −400–−80 kJ/mol for chemical desorption and −20–0 kJ/mol for physical desorption, with $\Delta H^{\theta }$ being >40 kJ/mol for chemical desorption and <40 kJ/mol for physical desorption. In the above three temperature conditions, the effects of hydrochloric acid on phosphorus desorption, the $\Delta G^{\theta }$ of physical adsorption, and the $\Delta H^{\theta }$ of chemical adsorption indicate that there is a chemical desorption of the desorption agent on the phosphorus of chemical and physical desorptions.

### 2.9. Desorption Kinetics

**Figure 10:**
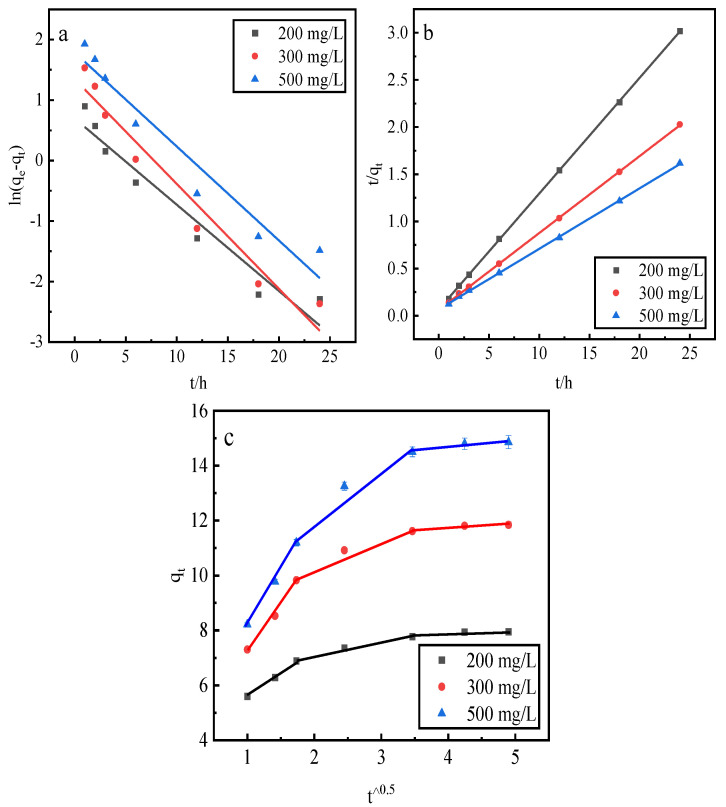
The fits of three desorption kinetic models: (**a**) the pseudo-first-order model, (**b**) the pseudo-second-order model, and (**c**) the intraparticle diffusion model.

This study employs a pseudo-first-order kinetic linear model, a pseudo-second-order kinetic linear model, and an intraparticle diffusion model to fit the phosphorus kinetic properties of adsorption by the desorbent desorption of activated red mud adsorbents. After adsorption with the different initial concentrations of 200, 300, and 500 mg/L, the activated red mud adsorbents were dried at 105 °C for 2 h. The desorbent was 0.2 mol/L HCl, and the desorbent and adsorbent solid–liquid ratio was 0.15 L/g. Static desorption was conducted at a constant temperature of 35 °C in conical flasks for 1, 2, 3, 6, 12, 18, and 24 h. The fitting results are presented in Figure 10 and Table 3.

Based on the correlation coefficient R^2^, the pseudo-second-order kinetic model exhibited better fitting for the phosphorus adsorption kinetics than the pseudo-first-order kinetic model, and the theoretical equilibrium detachment amounts were 15.073 mg/g at a concentration of 500 mg/L, 11.898 mg/g at a concentration of 300 mg/L, and 8.102 mg/g at a concentration of 200 mg/L. The theoretical equilibrium detachment amounts obtained are close to the experimental result *q_e_*, indicating that the chemical reaction limits the rate of surface desorption. The calculated *q_e_* is greater than the actual desorption maximum, which is also related to precipitation arising from the chemical reaction on the red mud surface. According to the intraparticle diffusion model, three main stages are observed: the first stage is the rapid desorption stage, which is primarily manifested in the form of the chemical precipitation of metal ions and phosphorus and the reaction occurring in the desorption agent. The second stage is slower and primarily involves the activation of the well-developed pore structure of the red mud to perform desorption. In the third stage, which is the slowest desorption, a relative equilibrium is gradually achieved. Because the internal diffusion curve of the particles does not pass through the origin of the coordinates, a high-concentration results in a greater deviation of the curve from the origin, indicating that internal diffusion is not the main mechanism of phosphorus desorption. Rather, desorption is dominated by the chemical reaction, in which phosphorus is removed through precipitation on the adsorbent surface of the activated red mud particles.

### 2.10. Surface Morphology Analysis after Desorption

**Figure 11:**
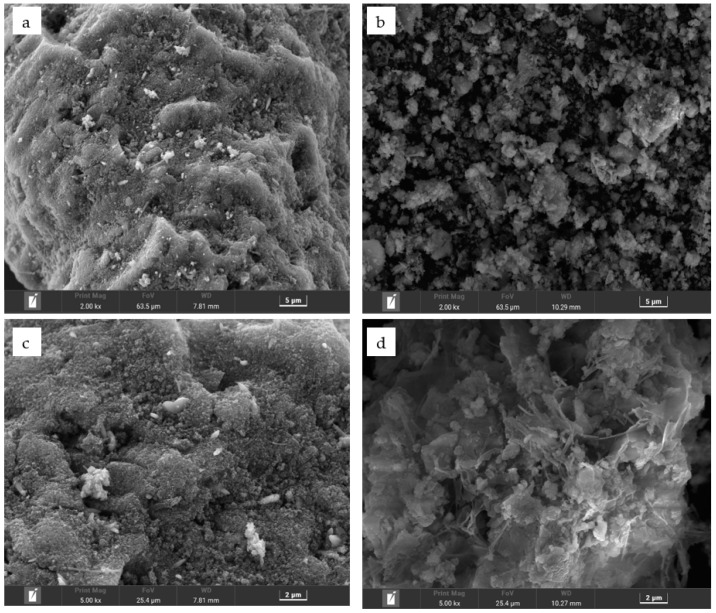
Scanning electron micrographs of the surface of activated red mud adsorbents before and after desorption. (**a**,**b**) Before and after desorption at 5 μm, respectively. (**c**,**d**) Before and after desorption at 2 μm, respectively.

Scanning electron microscopy (SEM) was used to observe the surface morphology of the activated particle adsorbents before (Figure 11a) and after (Figure 11b) desorption at 10,000× magnification and as well as before (Figure 11c) and after (Figure 11d) desorption at 25,000× magnification. The pores on the surface of the activated red mud adsorbent before desorption are covered by the precipitate, showing crystallization, multilayer adsorption on the surface of the activated red mud adsorbent, and precipitation products covering the material surface. The surface of the adsorbent after the desorption of the red mud adsorbent revealed notable corrosion after the destruction of the particle structure. To a certain extent, owing to the acid making the particles sparse in the pores, the effective points inside the particle pores were exposed. Figure 11c,d shows that the surface after desorption by the desorbent exhibits a laminated structure, which indicates surface disintegration, suggesting that the desorption of phosphorus is primarily caused by the particles disintegrating easily under the strong acid conditions of the desorbent.

**Figure 12:**
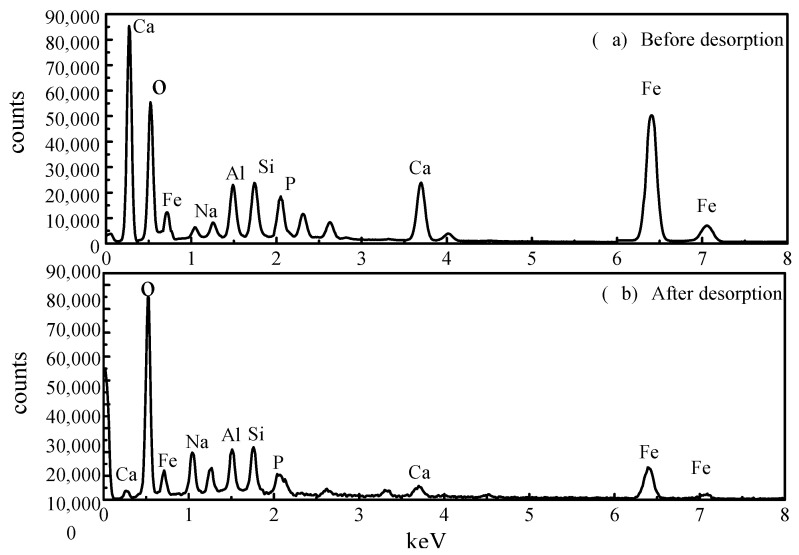
The narrow sweep of EDS before and after the desorption of activated red mud adsorbents.

Comparing the EDS measurements before and after the desorption of the activated red mud adsorbents reveals that the desorbent may have led to the disintegration of the adsorbent, as observed through SEM imaging. To explore the changes of the surface elements, EDS analyses were performed before and after desorption of the activated red mud adsorbents, and the surface of the particulate adsorbent before and after desorption was subjected to a narrow sweep of elements such as O, Ca, Al, Na, Si, Fe, and P (Figure 12). Figure 12a presents the narrow sweep of EDS before desorption, and Figure 12b shows this after desorption.

A comparison between Figure 12a,b and Table 4 shows that the elemental phosphorus on the surface of the adsorbent decreases after desorption, which is consistent with the SEM surface images presented in Figure 11. Less elemental calcium appeared on the surface of the granular adsorbents after desorption than before desorption, indicating that the dissolution of Ca in the activated red mud adsorbents under acidic conditions was linked to the reaction between the desorbent and the (Ca)_3_(PO_4_)_2_ precipitation on the adsorbent surface, causing certain phosphorus desorption. In addition, the specific surface elements before and after the desorption of phosphorus from the adsorbent were measured, with the results expressed in terms of elemental mass. The phosphorus adsorbed by the adsorbent on activated red mud particles after desorption decreased from 4.84% to 0.84%, Ca decreased from 15.54% to 8.97%, Al decreased from 6.47% to 5.48%, and Fe decreased from 13.65% to 12.65%, which also demonstrated that the desorbent reacted with the reactive elements in the red mud to cause the adsorption of the adsorbent on the surface by the active elements on the adsorbent surface. This result indicated that the desorbent reacted with the active elements in red mud, removing phosphorus originally adsorbed on the adsorbent surface. This was especially the case for the considerable reduction in Ca, which may react with the Cl^−^ in the desorbent to form water-soluble CaCl_2_. The corrosive effect caused these elements to detach from the surface of the activated red mud particles dissolved in the water and accompanied by phosphorus desorption.

### 2.11. Analysis of Morphological Changes

**Figure 13:**
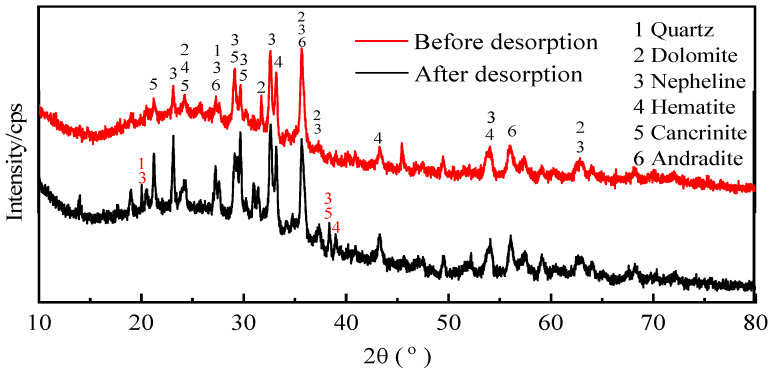
The XRD patterns of the activated red mud particles before and after desorption.

Figure 13 shows the mineral composition of the activated red mud adsorbent before and after desorption. With desorption, the intensities of the characteristic peaks of the chalcopyrite, calcium chalcopyrite, and calcium–iron garnet considerably decreased. This result is related to the disintegration and dissolution of elements such as Ca, Fe, and Si during phosphorus desorption in water. Variations in the crystal type and mineral composition are inconsiderable, indicating that the activated red mud adsorbents can continue to adsorb phosphorus after desorption. Thus, the adsorbents may be reused.

### 2.12. Fourier-Transform Infrared Spectroscopy Analysis

**Figure 14:**
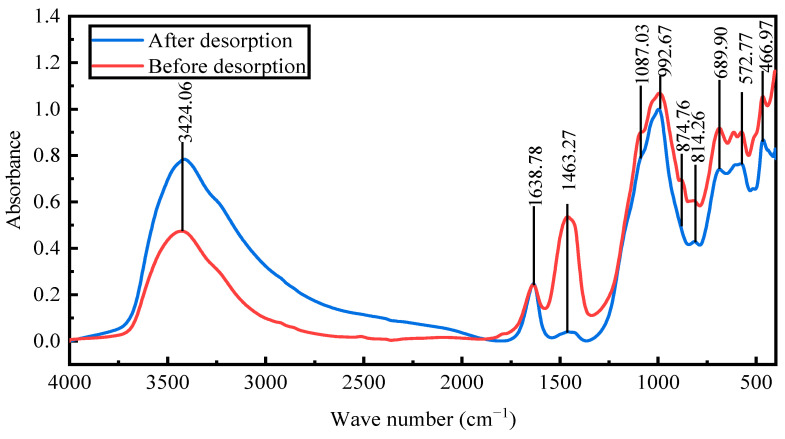
The Fourier-transform infrared spectra of activated red mud adsorbents before and after desorption.

Fourier-transform infrared spectroscopy was conducted to examine the changes in the structure and chemical groups of the activated red mud adsorbent with desorption. The goal was to verify the phosphorus fugacity state on the activated hematite particulate adsorbent from 400 to 1500 cm^−1^. Figure 14 presents the spectral maps before and after desorption. After desorption, the peaks at 992 cm^−1^ were considerably strengthened primarily by the Si(AlIV)–O telescopic vibration as shelly silicate or chalcocite, which was consistent with the increase in the Si content (EDS results). The peaks at 814, 874.76, and 1463 cm^−1^ disappeared, the main desorption agent led to the dissociation of Ca and Fe, and the vibrations of the [SiO_4_]^4−^ functional groups of the calcium–iron garnet and calcite or aragonite basically disappeared. The peaks at 3600–3000 cm^−1^ were due to water crystallization, indicating that desorption was accompanied by water desorption. Overall, the material changes before and after desorption were clearer than before desorption, especially for Ca and Fe compounds. After desorption, the intensities of the strong peaks for PO_4_^3−^ groups at 1087.03 and 572 cm^−1^ substantially decreased [22], indicating that the desorption of phosphorus from the activated red mud adsorbents was accompanied by a chemical reaction.

## 3. Materials and Methods

### 3.1. Materials

The raw materials for preparing modified red mud adsorbents primarily included red mud, charcoal powder, and silica sol. Red mud was procured from Guizhou Huajin Aluminum Co., Ltd., Guiyang, China, with the water content being 30%. It was dried at 50 °C for 12 h and then passed through a 0.075 mm sieve after grinding in a ball mill. Charcoal powder was procured from Henan Xingnuo Environmental Protection Materials Co., Ltd. (Changge, China), and silica sol was purchased from Wuhan Jiye Sheng Chemical Co. (Wuhan, China). The chemical components of the red mud were Fe_2_O_3_ (21.94%), Al_2_O_3_ (21.04%), SiO_2_ (19.05%), CaO (17.95%), Na_2_O (8.96%), and others (11.06%). The used phosphorus-containing wastewater was return water from a phosphorous-ore-dressing plant in Guizhou, China, and contained flotation tailings. Red mud particles were activated for use as adsorbents by mixing red mud, charcoal powder, and silica sol in a preset mass ratio of 92:5:3 by employing a disk granulator for granulation; the water–ash ratio was 1:2. These particles of 1–2 mm diameter were sintered in a muffle furnace at 700 °C for 30 min. The primary elements in the wastewater were P (1278.0 mg/L), Ca (213.15 mg/L), Mg (401.40 mg/L), Al (0.25 mg/L), Fe (0.02 mg/L), Cr (0.04 mg/L), K (66.35 mg/L), and Na (0.25 mg/L). The wastewater was weakly acidic (pH = 3–4); phosphorus was primarily present in the form of H_2_PO_4_^2−^, which could be diluted to the required concentration by adding deionized water.

### 3.2. Test Methods for Phosphorus Adsorption and Desorption Using and from, Respectively, Activated Red Mud Particles

The initial pH was 4. The effects of the operating and environmental conditions on the adsorption of the adsorbents were investigated as functions of the adsorbent dosage, adsorption time, and adsorption temperature to obtain the optimal adsorption conditions.

The initial phosphorus concentrations were 200, 250, 300, 400, 500, 600, 700, 900, and 1200 mg/L, the adsorption temperatures were 15 °C, 20 °C, 25 °C, 30 °C, and 35 °C, and the adsorption times were 1, 2, 3, 6, 12, 18, and 24 h. Further, 40 mL of phosphorus-containing wastewater was poured into a conical flask, and 0.8, 1, 1.2, 1.4, 1.6, 1.8, 2, 2.2, and 2.4 g of granular adsorbents were added for static adsorption. The test was conducted thrice using a model ICP-7400 inductively coupled plasma emission spectrometer and a yttrium internal standard to measure the contents of phosphorus and other ions in the supernatant. The phosphorus removal rate (η%) and the amount of phosphorus adsorbed per unit adsorbent mass (*Q*, mg/g) were calculated as follows [23]:

(1) $$\eta =\frac{\left(C_{0}-C_{e}\right)}{C_{0}}\times 100\%,$$

(2) $$Q=\frac{V(C_{o}-C_{e})}{m}\times 100\%,$$

where $C_{o}$ (mg/L) and $C_{e}$ (mg/L) are the concentrations of phosphorus in the used solution before and after adsorption, respectively, *V* is the solution volume (L), and *M* is the mass of the adsorbent after drying (g).

*Phosphorus desorption test.* Activated red mud adsorbents were removed and dried and then added into different types of desorbents. Acid: 0.2, 0.1, and 0.05 mol/L HCl; alkali: 0.5, 1, and 2 mol/L NaOH; and salt: 1 and 2 mol/L NaCl. The desorption temperatures were 15 °C, 20 °C, 25 °C, 30 °C, and 35 °C, and the desorption times were 1, 2, 3, 6, 12, 18, and 24 h. The concentration of phosphorus desorbed from the solution was determined at the end of the test. The amount of phosphorus desorbed per unit adsorbent mass after adsorption (*Y*, mg/g) and the phosphorus desorption rate (*y*, %) were calculated using Equations (4) and (5) [24]:

(3) $$q_{e}=(C_{0}-C_{e})\frac{V_{1}}{m_{1}},$$

(4) $$Y=C_{p}\frac{V_{2}}{m_{2}},$$

(5) $$y=\frac{Y}{q_{e}}\times 100\%,$$

where $q_{e}$ is the amount of phosphorus adsorbed per unit adsorbent mass (mg/g), $C_{0}$(mg/L) and $C_{e}$ (mg/L) are the concentrations of phosphate in the adsorption solution before and after the test, respectively, $V_{1}$ is the adsorption solution volume (L), $m_{1}$ is the added adsorbent mass (g), $C_{p}$ is the concentration of phosphorus in the desorption solution (mg/L), $V_{2}$ is the volume of the desorption solution (L), and $m_{2}$ is the dry weight of the adsorbents after adsorption (g).

*Phosphorus adsorption test.* After desorption, the activated red mud adsorbents were first rinsed with deionized water and then dried to be used again for phosphorus adsorption. To obtain the optimal adsorption conditions for any other pharmaceutical compound, the steps are the same as those for the activated red mud adsorbents.

### 3.3. Characterization and Analysis Methods

The specific surface area and porosity were determined using a specific surface area and porosity analyzer model (Micromeritics APSP 2460, Norcross, GA, USA), with a degassing temperature of 200 °C, a degassing time of 8 h, and nitrogen adsorption gas. The specific surface area and pore volume of the red mud samples were calculated before and after activation using the Brunauer–Emmett–Teller (BET) and Barrett–Joyner–Halenda (BJH) models.

The elemental content and composition of red mud before and after the test were analyzed using an ARL PERFORM’X X-ray (Basel, Switzerland) fluorescence spectrometer. The red mud to be tested was dried, ground, and filtered using a 200 mesh; 4 g of the sample was weighed and analyzed in a vacuum.

Thermogravimetric analysis was performed using synchronous PE thermogravimetry–differential scanning calorimetry to determine the activation temperature by analyzing the changes in the masses of the charcoal powder and red mud as functions of temperature. The heating rate was 10 °C/min, the temperature range was 30 °C–1000 °C, and the atmosphere was nitrogen.

Surface micromorphological analysis and energy spectroscopy were performed using a TESCAN MIRA LMS scanning electron microscope (Brno, Czech Republic) to image and analyze the surface configuration of the red mud adsorbents. The morphology was enlarged to 2–5 μm, and the particle surfaces were scanned through EDS in the spectral spot scanning mode to analyze the relative contents of phosphorus and other elements on the sample surface.

Mineral composition analysis was performed using an X-ray diffractometer model (XPert PRO MPD, Almelo, The Netherlands) to determine the mineral composition of the samples through powder X-ray diffraction (operating parameters: Cu Kα line; 40 kV and 40 mA; scanning speed = 2°/s; and scanning range = 10°–80°).

The surface functional groups were analyzed using a Fourier infrared spectrometer model (Nicolet 670, Waltham, MA, USA). The position and intensity of the Fourier infrared absorption peaks reflect the characteristics of the molecular structure and serve to identify the structural composition of the unknown substance or to determine its chemical composition. For infrared analysis of the bulk, a small amount of the dried sample was mixed with potassium bromide powder that was ground and pressed in a vacuum. The functional groups were then identified via infrared spectral scanning from 400 to 4000 cm^−1^.

### 3.4. Kinetic and Thermodynamic Methods

#### 3.4.1. Desorption Isotherm Analysis

Desorption curves at different temperatures are nonlinear and can, therefore, be fitted using the Freundlich and Langmuir adsorption isotherm equations [25]. The isotherms reveal the interaction between (i) the adsorbent and desorbent and (ii) the adsorbate at the interface of the two phases to obtain the desorption properties and mechanism [26]. The D–R model provides the ideal state, assuming that the adsorbent pore space is filled with solute. The Freundlich adsorption isotherm model (Equation (6)) and the Langmuir adsorption isotherm model (Equation (7)) were employed to analyze the adsorption isotherms.

(6) $$q_{e}=K{C_{e}}^{1/n},$$

(7) $$q_{e}=\frac{abC_{e}}{1+bC_{e}},$$

(8) $$lnq_{e}=lnq_{m}-k\varepsilon^{2},$$

where *K* is the Freundlich constant of adsorption and desorption, the exponent 1/n is the Freundlich affinity coefficient for the adsorption–desorption intensity, *a* and *b* are the Langmuir adsorption–desorption capacity and binding strength, respectively, $C_{e}$ is the phosphate concentration at adsorption–desorption equilibrium, $q_{e}$ is the equilibrium adsorption amount (mmol/g), $q_{m}$ is the theoretical maximum adsorption amount (mmol/g), k and ε are constants, and $E=-1/{\left(2k\right)}^{0.5}$.

#### 3.4.2. Desorption Thermodynamic Analysis

Thermodynamics is employed from the energy point of view to explain a possible desorption mechanism, explore the energy conversion law, and use thermodynamic parameters to understand the characteristics of the following thermodynamic equations [27]:

(9) $$K_{d}=q_{e}/C_{e},$$

(10) $$\Delta G^{\theta }=-RT\ln K_{d},$$

(11) $$\Delta G^{\theta }=\Delta H^{\theta }-T\Delta S^{\theta },$$

where $\Delta G^{\theta }$ is the change in the Gibbs free energy, $K_{d}$ is a thermodynamic constant, $\Delta H^{\theta }$ is the change in enthalpy, $\Delta S^{\theta }$ is the change in entropy, R is the universal gas constant, and *T* is the absolute temperature. Combining Equations (10) and (11) results in the following equation:

(12) $$\ln K_{d}=\frac{\Delta S^{\theta }}{R}-\frac{\Delta H^{\theta }}{RT}.$$

#### 3.4.3. Analysis of Desorption Kinetics

Desorption kinetic models were employed to evaluate the adsorption–desorption behavior of red mud for phosphate as a function of the reaction time [28]. Ruthven used an adsorption dynamics model to calculate and investigate the relation between desorption and adsorption penetration curves, indicating the difference between the two based on the equilibrium theory applied to desorption [29]. The kinetic models used in this study are based on a pseudo-first-order kinetic linear model (Equation (13)) [29], a pseudo-second-order kinetic linear model (Equation (14)) [30], and an intraparticle diffusion model (Equation (15)) [31,32] to fit the following kinetics of phosphate adsorption by activated red mud adsorbent:

(13) $$q_{t}=q_{e}-(q_{e}\times 10^{\frac{-k_{1}t}{2.303}}),$$

(14) $$\frac{t}{q_{t}}=\frac{1}{k_{2}{q_{e}}^{2}}+\frac{t}{q_{e}},$$

(15) $$q_{t}=k_{i}t^{1/2}+C,$$

where *k*_1_ and *k*_2_ are the rate constants of the pseudo-first-order and pseudo-second-order kinetic linear models, respectively, *k_i_* is the intraparticle diffusion constant, *q_t_* is the amount of phosphate desorbed by the adsorbent in the desorption time (h), and *C* is the amount of diffusion.

## 4. Conclusions

(1) For static desorption, the comprehensive desorption effect may be ranked as HCl > NaOH > NaCl > deionized water. The higher the desorbent concentration, the greater the desorption and the better the desorption of 0.2 mol/L of HCl in resorption. Regeneration improves owing to acids, which clean the pores of red mud particles, thereby exposing the effective sites inside the pores.
(2) The desorption thermodynamics and kinetics show that desorption conforms to the average-adsorption-free-energy reaction obtained by the D–R model. The desorption isotherm correlates well with the Langmuir model, indicating that desorption is dominated by monomolecular-layer surface desorption. The thermodynamics show that the desorption of phosphorus by the desorbents proceeds spontaneously, and high temperatures promote desorption. Desorption thus includes both physical and chemical desorptions.
(3) The kinetics indicate that desorption conforms to the pseudo-second-order kinetic linear simulation, indicating that chemical desorption restricts the desorption rate. The intraparticle diffusion model indicates that the desorbent removes phosphorus in the form of precipitation from the surface of the activated red mud adsorbent primarily through a chemical reaction.
(4) Microanalysis before and after the desorption of the desorbent and surface elemental analysis demonstrate that P is removed and that Ca is dissolved the most, followed by Al and Fe, indicating that the desorbents react with the active elements in red mud. The corrosive effect leads to the disintegration and dissolution of the elements at the surfaces of the activated red mud adsorbents in water. This is accompanied by phosphorus desorption; hence, the characteristic peak intensities of chalcopyrite, calcite chalcopyrite, and calcite garnet decrease more than that of the activated red mud adsorbents. After desorption, the mineral composition remains similar because adsorbent reuse has a negligible impact on adsorption. The vibrations of the [SiO_4_]^4−^ functional groups of calcium–iron garnet and calcite or aragonite disappear, the PO_4_^3−^ group peak intensities substantially decrease, and the desorption of phosphorus is accompanied by a chemical reaction.

## Figures and Tables

**Table 1:** Desorption isotherm parameters.

Desorption Isotherm	Constants		
	308 K	298 K	288 K
**Langmuir**	R = 0.9947 a (mg/g) = 19.4175 b (L/mg) = 1.8392	R = 0.9946 a (mg/g) = 19.3798 b (L/mg) = 1.0639	R = 0.9950 a (mg/g) = 19.3050 b (L/mg) = 0.7674
**Freundlich** **D–R**	R = 0.9400 N = 4.7393 K_F_ (mg^1−n^/g L^n^) = 12.0637 R = 0.9916 q_m_ (mmol/L) = 0.03255 k (mol^2^/kJ^2^) = 0.0005 E (kJ/mol) = −31.6228	R = 0.9118 n = 4.8852 K_F_/(mg^1−n^/g L^n^) = 10.8038	R = 0.9438 n = 5.0684 K_F_/(mg^1−n^/g L^n^) = 10.1808

**Table 2:** The thermodynamic parameters of phosphorus desorption from activated red mud particle sorbents.

T (K)	$\Delta G^{\theta }$ (kJ/mol)	$\Delta H^{\theta }$ (kJ/mol)	$\Delta S^{\theta }$ (J/(mol⋅K))	$R^{2}$
288	−14.4085			
298	−16.0027	42.7173	197.9314	0.9755
308	−18.3846			

**Table 3:** Desorption kinetic parameters.

Adsorption Kinetics	Constant		500 mg/L	300 mg/L	200 mg/L
Pseudo-first-order dynamic linear model	q_e_ (mg/g)		5.9817	3.8551	2.0100
	k_1_ (1/h)		0.1554	0.1734	0.1427
	R^2^		0.9438	0.9537	0.9433
Pseudo-second-order dynamic linear model	q_e_ (mg/g)		15.5763	12.2549	8.1499
	k_2_ (1/h)		0.0613	0.1102	0.2221
	R^2^		0.9998	0.9999	0.9999
Intraparticle diffusion model	Phase I	k_i_ (mg/g h^1/2^)	3.9826	1.7320	3.2657
		C (mg/g)	4.2189	3.8600	4.0257
		R^2^	0.9978	0.9984	0.9929
	Phase II	k_i_ (mg/g·h^1/2^)	1.9701	0.5040	1.0750
		C (mg/g)	7.9605	6.0442	8.0460
		R^2^	0.9462	0.9889	0.9525
	Phase III	k_i_ (mg/g·h^1/2^)	0.2629	0.1053	0.1749
		C (mg/g)	13.6168	7.4382	11.0230
		R^2^	0.9138	0.8535	0.9120

**Table 4:** The elemental composition mass fraction (%) of activated red mud adsorbents before and after desorption.

Element	Before Desorption (%)	After Desorption (%)
O	51.04	61.03
Na	2.21	3.84
Al	6.47	5.48
Si	6.25	7.22
P	4.84	0.84
Ca	15.54	8.97
Fe	13.65	12.62
Total	100.00	100.00

## Data Availability

Data are contained within the article.

## References

[1] Chen W. (2010). Exploratory study on the preparation of modified red mud porous material and wastewater treatment. J. China Univ. Geosci..

[2] Wang Y.B., Zhu X.F., Zhang X.L. (2010). Removal of high concentration phosphorus wastewater by red mud. Chem. Eng. Prog..

[3] Azizian S. (2004). Kinetic models of sorption: A theoretical analysis. J. Colloid Interf. Sci..

[4] Xing X., Gao B., Yue Q., Zhong Q. (2011). Sorption of phosphate onto giant reed based adsorbent: FTIR, Raman spectrum analysis and dynamic sorption/desorption properties in filter bed. Bioresour. Technol..

[5] Johir M.A.H., George J., Vigneswaran S., Kandasamy J., Grasmick A. (2011). Removal and recovery of nutrients by ion exchange from high rate membrane bio-reactor (MBR) effluent. Desalination.

[6] Park K.Y., Song J.H., Lee S.H., Kim H.S. (2010). Utilization of a selective adsorbent for phosphorus removal from wastewaters. Environ. Eng. Sci..

[7] Cheng X., Huang X., Wang X., Zhao B., Chen A., Sun D. (2009). Phosphate adsorption from sewage sludge filtrate using zinc–aluminum layered double hydroxides. J. Hazard. Mater..

[8] Urano K., Tachikawa H. (1991). Process development for removal and recovery of phosphorus from wastewater by a new adsorbent. 1. Preparation method and adsorption capability of a new adsorbent. Ind. Eng. Chem. Res..

[9] Delaney P., McManamon C., Hanrahan J.P., Copley M.P., Holmes J.D., Morris M.A. (2011). Development of chemically engineered porous metal oxides for phosphate removal. J. Hazard. Mater..

[10] Urano K., Tachikawa H. (1992). Process development for removal and recovery of phosphorus from wastewater by a new adsorbent. 3. Desorption of phosphate and regeneration of adsorbent. Ind. Eng. Chem. Res..

[11] Ye J., Cong X., Zhang P., Zeng G., Hoffmann E., Liu Y., Hahn H.H. (2016). Application of acid-activated Bauxsol for wastewater treatment with high phosphate concentration: Characterization, adsorption optimization, and desorption behaviors. J. Environ. Manag..

[12] Zhao Y., Yue Q., Li Q., Gao B., Han S., Yu H. (2010). The regeneration characteristics of various red mud granular adsorbents (RMGA) for phosphate removal using different desorption reagents. J. Hazard. Mater..

[13] Zhang G., Liu H., Liu R., Qu J. (2009). Removal of phosphate from water by a Fe–Mn binary oxide adsorbent. J. Colloid Interf. Sci..

[14] Zeng L., Li X., Liu J. (2004). Adsorptive removal of phosphate from aqueous solutions using iron oxide tailings. Water Res..

[15] Ajmal Z., Muhmood A., Usman M., Kizito S., Lu J., Dong R., Wu S. (2018). Phosphate removal from aqueous solution using iron oxides: Adsorption, desorption and regeneration characteristics. J. Colloid Interf. Sci..

[16] Xie W.M., Zhou F.P., Bi X.L., Chen D.D., Li J., Sun S.Y., Liu J.-Y., Chen X.-Q. (2018). Accelerated crystallization of magnetic 4A-zeolite synthesized from red mud for application in removal of mixed heavy metal ions. J. Hazard. Mater..

[17] Kuzawa K., Jung Y.J., Kiso Y., Yamada T., Nagai M., Lee T.G. (2006). Phosphate removal and recovery with a synthetic hydrotalcite as an adsorbent. Chemosphere.

[18] Li Y., Liu C., Luan Z., Peng X., Zhu C., Chen Z., Zhang Z., Fan J., Jia Z. (2006). Phosphate removal from aqueous solutions using raw and activated red mud and fly ash. J. Hazard. Mater..

[19] Zhao Y.Q. (2013). Preparation and Characterisation of New Red Mud Particle Adsorbent Material and Its Performance on Phosphorus Removal from Water Body.

[20] Xu M.Y. (2020). Preparation, Characterisation and Properties of Red Mud-Based Granular Phosphorus Removal Materials.

[21] Wu H.L., Wei S.N., Cui S.L. (2006). Introduction and application of adsorption isotherms. Dye. Finish. Technol..

[22] Pepper R.-A., Couperthwaite S.-J., Millar G.-J. (2018). Re-use of waste red mud: Production of a functional iron oxide adsorbent for removal of phosphorous. J. Water Process Eng..

[23] Huang Q.B. (2018). Research on the Removal of Nitrate and Phosphate in Water by Adsorption of Organically Modified Aluminium-Manganese Composite Oxides.

[24] Wu J. (2016). Preparation of Red Mud Base Polymer Porous Material and Its Adsorption Performance.

[25] Jiang Y., Ma X.L., Guo Y.X., Sun F.Y., Sun J., He X.Y. (2021). Adsorption and desorption characteristics of zinc in different layered soils. J. Northeast. Univ..

[26] Deihimi N., Irannajad M., Rezai B. (2018). Equilibrium and kinetic studies of ferricyanide adsorption from aqueous solution by activated red mud. J. Environ. Manag..

[27] Zhu C., Luan Z., Wang Y., Shan X. (2007). Removal of cadmium from aqueous solutions by adsorption on granular red mud (GRM). Sep. Purif. Technol..

[28] Xia L.Y. (2021). Effects of pH and organic matter on the adsorption-desorption characteristics of zinc in soil. Energy Environ. Protection..

[29] Yao H.Q., Liu X.Q., Ma Z.F. (1990). Kinetics of water desorption on zeolite. J. Nanjing Univ..

[30] Ahmed M.J., Dhedan S.K. (2012). Equilibrium isotherms and kinetics modeling of methylene blue adsorption on agricultural wastes-based activated carbons. Fluid Phase Equilibria.

[31] Cheung W.H., Szeto Y.S., McKay G. (2007). Intraparticle diffusion processes during acid dye adsorption onto chitosan. Bioresour. Technol..

[32] Weber W.J., Morris J.C. (1963). Kinetics of adsorption on carbon from solution. J. Sanit. Eng. Div..

